# Advanced organ support (ADVOS) in the critically ill: first clinical experience in patients with multiple organ failure

**DOI:** 10.1186/s13613-020-00714-3

**Published:** 2020-07-16

**Authors:** Valentin Fuhrmann, Theresa Weber, Kevin Roedl, Jasmin Motaabbed, Adel Tariparast, Dominik Jarczak, Aritz Perez Ruiz de Garibay, Johannes Kluwe, Olaf Boenisch, Harald Herkner, John A. Kellum, Stefan Kluge

**Affiliations:** 1 Department of Intensive Care Medicine, University Medical Center Hamburg-Eppendorf, Martinistraße 52, 20246 Hamburg, Germany; 2 Department of Medicine B, University Münster, Münster, Germany; 3 University of Strasbourg, CNRS, Immunopathology and Therapeutic Chemistry, UPR 3572, 67000 Strasbourg, France; 4 Department of Internal Medicine 1, University Medical Center Hamburg-Eppendorf, Hamburg, Germany; 5 Department of Critical Care Medicine, Center for Critical Care Nephrology, University of Pittsburgh Medical Center, Pittsburgh, USA; 6 Department of Emergency Medicine, Medical University Vienna, Vienna, Austria

**Keywords:** Multiple organ failure, Extracorporeal organ support, Albumin dialysis, Acute liver failure, Acidosis, ARDS, Septic shock

## Abstract

**Background:**

Prevalence of multiple organ failure (MOF) in critically ill patients is increasing and associated mortality remains high. Extracorporeal organ support is a cornerstone in the management of MOF. We report data of an advanced hemodialysis system based on albumin dialysis (ADVOS multi device) that can regulate acid–base balance in addition to the established properties of renal replacement therapy and albumin dialysis systems in critically ill patients with MOF.

**Methods:**

34 critically ill patients with MOF received 102 ADVOS treatment sessions in the Department of Intensive Care Medicine of the University Medical Center Hamburg-Eppendorf. Markers of metabolic detoxification and acid–base regulation were collected and blood gas analyses were performed. A subgroup analyses were performed in patients with severe acidemia (pH < 7.2).

**Results:**

Median number of treatment sessions was 2 (range 1–9) per patient. Median duration of treatment was 17.5 (IQR 11–23) hours per session. Treatment with the ADVOS multi-albumin dialysis device caused a significant decrease in bilirubin levels, serum creatinine, BUN and ammonia levels. The relative elimination rate of bilirubin was concentration dependent. Furthermore, a significant improvement in blood pH, HCO_3_^−^ and PaCO_2_, was achieved during ADVOS treatment including six patients that suffered from severe metabolic acidosis refractory to continuous renal replacement therapy. Delta pH, HCO_3_^−^ and PaCO_2_ were significantly affected by the ADVOS blood flow rate and pH settings. This improvement in the clinical course during ADVOS treatments allowed a reduction in norepinephrine during ADVOS therapy. Treatments were well tolerated. Mortality rates were 50% and 62% for 28 and 90 days, respectively.

**Conclusions:**

In this case series in patients with MOF, ADVOS was able to eliminate water-soluble and albumin-bound substances. Furthermore, the device corrected severe metabolic and respiratory acid–base disequilibrium. No major adverse events associated with the ADVOS treatments were observed.

## Background

Frequency of multiple organ failure (MOF) is significantly increasing in critically ill patients within the last decades with a prevalence of more than 30% [1]. Despite significant progress in management, mortality in patients with advanced stages of MOF is still excessively high. Recent publications indicate that more than 60% of these patients did not survive their stay at the ICU [1, 2]. Liver failure is present in 20% of the ICU patients [1]. Acute kidney injury (AKI) occurs in > 50% of critically ill patients [3] and is more common in association with various forms of liver disease [4–6]. AKI contributes significantly to increased morbidity and mortality in these patients [5, 7, 8]. Respiratory failure is present in up to 30 percent of patients with acute liver failure during their stay at the ICU [9, 10].

In recent years, extracorporeal support has become common place in the management of patients with liver failure [11]. These treatments are usually indicated as a bridge to recovery or transplantation in critically ill patients with various kinds of organ failure. Although conventional renal replacement modalities are commonly used during daily clinical practice in patients with liver failure, most published data are available for advanced dialysis devices like liver support systems [12–14]. However, recommendations from professional societies note that using extracorporeal therapies in patients with liver failure is controversial and clinical practice is variable [15–17].

As suggested by Bellomo and Ronco already in the 1990s, a device combining different forms of organ support might be feasible to improve patient outcome in patients with MOF [18]. This concept was further described by Ranieri et al. defining the term extracorporeal organ support (ECOS) to represent all forms of devices, encompassing kidney, respiratory, cardiac and liver support [19].

ADVanced Organ Support (ADVOS, ADVITOS GmbH, Munich, Germany) is a new albumin dialysis procedure (CE-Marking obtained on 2013) that can eliminate water-soluble and albumin-bound substances. Furthermore, it can correct acid–base abnormalities by an adjustable dialysate composition on an individual basis. ADVOS uses a novel system for dialysate recirculation where physicochemical changes (i.e., pH and temperature) are implemented. Apart from properties of a conventional dialysis device, this allows for (i) recycling albumin following a modification of the pH in the albumin circuit of the device resulting in a conformational change of albumin that contributes to toxin release and liberation of binding sites [20]; (ii) customization of dialysate acid–base composition (including pH) as the dialysate is formed via the on-line mixing of an acidic and an alkaline concentrate, which can be automatically formulated to the patients’ needs in order to control acid–base balance. Preclinical studies demonstrated high detoxification rates for protein-bound and water-soluble toxins. Removal of relevant amounts of CO_2_ at blood flow rates typical for renal replacement therapies was shown in vitro [21–23]. A clinical case series using a prototype of the system demonstrated the ability to remove water-soluble and albumin-bound substances [24]. Here, we report our first clinical experience with ADVOS in the management of critically ill patients with severe MOF.

## Materials and methods

This analysis included data from 34 patients with MOF receiving 102 treatment sessions with the ADVOS device. All patients were treated as clinically indicated at the Department of Intensive Care Medicine of the University Medical Center Hamburg-Eppendorf on-label of the intended use of the device, as indicated in the instructions for use of the manufacturer. Patients were treated between December 2014 and August 2016. Data were automatically prospectively documented in the electronic patient data management system (ICM, Dräger, Lübeck, Germany) and thereafter extracted retrospectively for statistical analysis. Patients were followed up for 90 days after ICU admission, until ICU discharge or until death, whichever occurs first. The study was approved by the local ethics committee (WF 046/15).

On admission, Simplified Acute Physiology Score II (SAPS II) [25], Sequential Organ Failure Assessment (SOFA) Score [26], infections, organ failure and need for vasopressor support, mechanical ventilation or renal replacement therapy (RRT), were documented. Daily data from routine laboratory conducted analysis were documented.

Diagnostic criteria for AKI, hypoxic liver injury (HLI), cardiogenic shock and septic shock have been previously described [4]. In detail, criteria for AKI were calculated according to the KDIGO clinical practice guideline for AKI based on serum creatinine and urinary output [27]. All patients had AKI stage 3 prior to initiation of ADVOS and suffered from different forms of liver failure (acute-on-chronic liver failure, primary and secondary acute liver failure) [27]. Acute-on-chronic liver failure (ACLF) is defined by presence of acute hepatic decompensation in combination with hepatic or extrahepatic organ failure (as defined by the CLIF-consortium and the CLIF-SOFA-score) [7]. Acute liver failure (ALF) is defined according to the recent definition of EASL practical clinical guidelines by INR > 1.5 and presence of hepatic encephalopathy [28]. Hypoxic liver injury (HLI) was defined according to well-established criteria: (a) setting of cardiac, circulatory or respiratory failure; (b) dramatic but transient elevation of aminotransferase levels to at least 20-fold the upper limit of normal; (c) exclusion of other putative causes of liver cell necrosis (viral or drug induced hepatitis) [7, 29, 30]. ARDS was defined according to the Berlin definition [31]. ADVOS was started in patients with severe metabolic derangement, anuria irresponsive to fluids, hyperkalemia and/or uremic complications, as previously published [4, 32, 33].

Severe metabolic acidemia was defined as pH less than 7.2, partial pressure of carbon dioxide (PaCO_2_) ≤ 45 mmHg and sodium bicarbonate < 22 mmol/l according to previous publications [34, 35]. 28-day mortality, 90-day mortality and ICU mortality were assessed on site or by contacting the patient, respectively.

### ADVOS multi

**Fig. 1:**
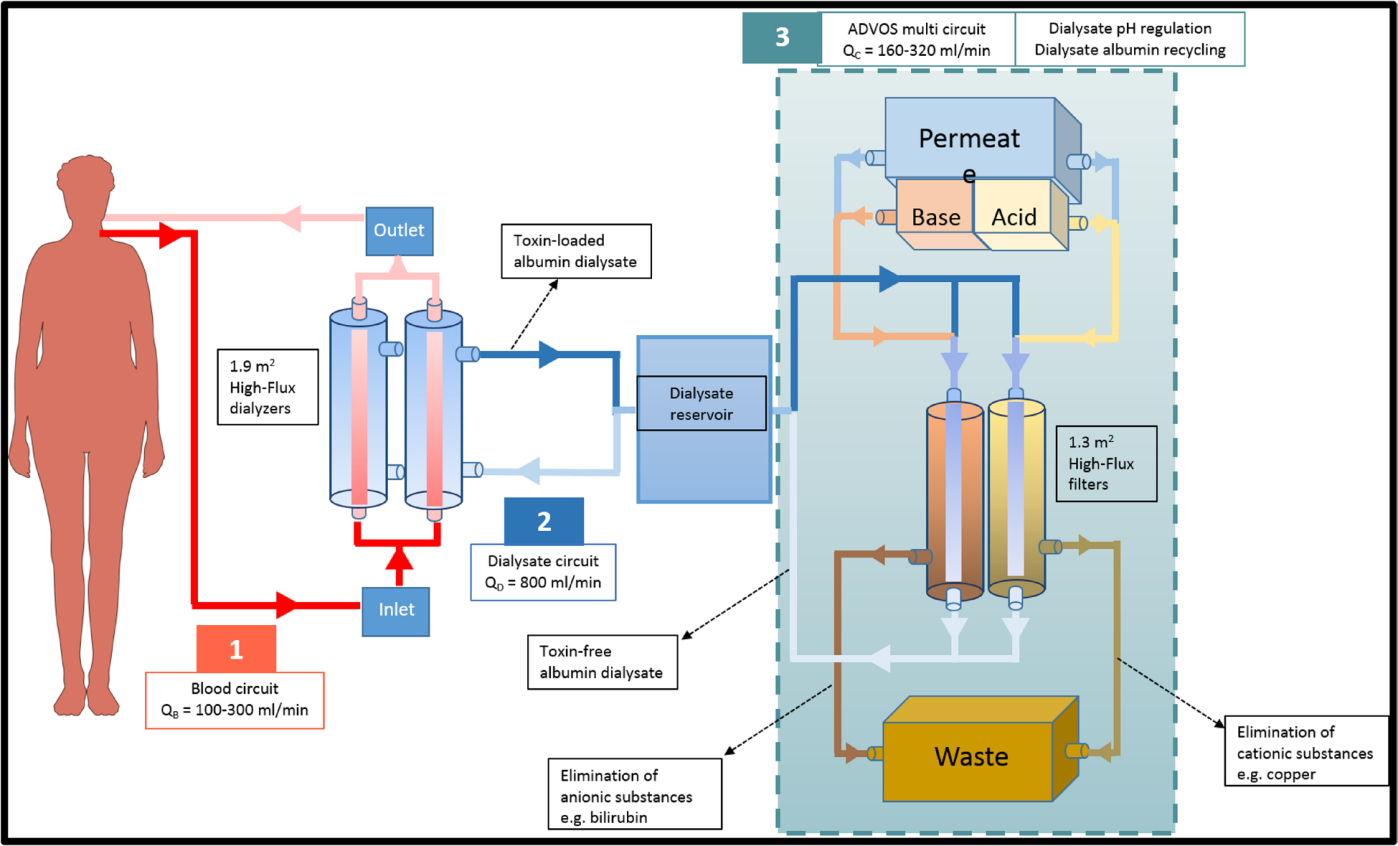
The ADVOS system

Patients were treated with the ADVOS multi device (ADVITOS GmbH, Munich, Germany), which is based on albumin dialysis that is connected to patients by a conventional double-lumen dialysis catheter. As previously described [24], the ADVOS system consists of 3 circuits: an extracorporeal blood circuit, a dialysate circuit and the ADVOS multi circuit. Figure 1 illustrates the detailed setting of the device. In the extracorporeal or blood circuit two high-flux polyethersulfone dialyzers (SURELYZER PES-190 DH. Nipro D.Med Germany GmbH, Hamburg, Germany) with a 1.9-m^2^ effective surface were perfused. This setting allows blood flows between 100 and 400 ml/min.

The dialysate and the ADVOS circuit together eliminate protein-bound and water-soluble toxins from the patient´s blood. Briefly, the dialysate contains 200 ml of 20% albumin and recirculates (dialysate flow of 800 ml/min) in the second circuit. The dialysate albumin provides binding sites for protein-bound toxins, whose unbound fraction can diffuse from blood through the semipermeable dialyzer membrane. In case of high concentration of toxins (bound and unbound), blood albumin binding sites will be occupied and a diffusion gradient of toxins into the dialysate could therefore be maintained.

Finally, the albumin´s binding capacity is restored by adding in parallel acidic and basic concentrates (concentrate flow between 160 and 320 ml/min) in the ADVOS multi circuit which results in the following dialysate concentrations (mmol/l): Na^+^ 133–145 mmol/l, Cl^−^ 100-106 mmol/l, K^+^ 2.8 mmol/l, Ca^2+^ 1.15-1.22, Mg^2+^ 0.5, HPO_4_^2−^ 0.5 and HCO_3_^−^ 25–26. 40% glucose (70 ml/h) is additionally administered into the dialysate through an accessory port. Likewise, toxins are released and filtered through two commercially available polynephron high-flux filters (ELISIO-13H. Nipro D.Med Germany GmbH. Hamburg, Germany) with an effective surface of 1.3 m^2^. Each filter is part of either the acidic or the alkaline path (Fig. 1), where due to pH differences cationic (e.g., copper) or anionic (e.g., bilirubin) toxins, respectively, might be released from albumin and filtered.

Compared to the prototype previously described [24], an additional automatic control was included in the current ADVOS generation that allows the modification of the dialysate acid–base composition. For this purpose, a pH value between 7.2 and 9.0 can be set for the dialysate, which provides an automatic adjustment of the dialysate composition according to the amount of each concentrate being supplied. In this setup, albumin, as opposed to bicarbonate, acts as the principal buffer. Briefly, according to quantitative acid–base balance theory [36–39], albumin is a weak acid, and allows to retain Na^+^ or Cl^−^, modifying the strong ion difference (SID) and altering dialysate pH. The higher the SID (and pH) in the dialysate, the higher the reduction of H^+^ concentration that can be achieved in blood. In this way, ADVOS can be thought of providing “renal compensation” of acidosis by shifting the CO_2_ equilibrium toward bicarbonate [40].

Anticoagulation was employed on clinical judgement. In detail, unfractioned heparin (UFH) was used in 44 treatment sessions, regional citrate anticoagulation (CiCa) in 45 treatment sessions, antithrombin III (AT III) in 10 treatment sessions and no anticoagulation was applied in 3 sessions.

### Statistical analysis

All continuous variables are reported as median and 25–75% interquartile range (IQR). Categorical variables were compared via Chi square analysis or Fisher’s exact, as appropriate. Metric variables were compared via Mann–Whitney *U*-test. Paired continuous variables were compared via Wilcoxon test. For correlations assessment Pearson’s coefficient was employed. SPSS 24 for Windows (SPSS, Inc. Chicago, IL) was used for statistical analysis. We calculated mean differences of post-therapy minus pre-therapy values. For the 102 observations in 34 patients we calculated between patient standard deviations. Using a random effects linear regression model, we estimated the patient-level mean difference together with a 95% confidence interval. By a Wald test, we tested the null hypothesis of the mean difference = 0. For the analyses we used Stata 14.0 (Stata Corp, College Station, TX). Generally, a two-sided *p* value < 0.05 was considered statistically significant.

## Results

### Patient characteristics

Thirty-four patients that were consecutively treated with the ADVOS device were included in this study. Median age was 59 years, 68% of the patients were male. Median SAPS II score was 52 (IQR 43–58) and median SOFA score was 17 (IQR 14–19). Twenty-six patients received mechanical ventilation and 25 patients were on vasopressors prior to initiation of ADVOS. The median interval between the ICU admission and the first ADVOS treatment was 3 days (IQR: 1–12). Detailed patients’ characteristics are illustrated in Table 1.

**Table 1:** Patients’ demographics (*n* = 34)

Parameter	Results
Male, *n* (%)	23 (68)
Age (years), median (IQR)	59 (46–72)
Size (cm), median (IQR)	178 (170–180)
Weight (kg), median (IQR)	75 (73–86)
SAPS 2, median (IQR)	52 (43–58)
SOFA, median (IQR)	17 (14–19)
Main admission diagnosis at the ICU	
Septic shock, *n* (%)	16 (47)
ARDS, *n* (%)	9 (26)
Cardiogenic shock, *n* (%)	4 (12)
Liver failure, *n* (%)	5 (15)
Etiology of liver failure	
Acute-on-chronic liver failure, *n* (%)	15 (44)
Acute liver failure, *n* (%)	19 (56)
Acquired acute liver failure, *n* (%)	15 (44)
Post-transplant liver failure, *n* (%)	3 (9)
Primary acute liver failure, *n* (%)	1 (3)
Treatment	
Vasopressor therapy, *n* (%)	25 (73)
Mechanical ventilation, *n* (%)	26 (76)
Renal replacement therapy, *n* (%)	34 (100)
Outcome	
ICU-LOS (days), median (IQR)	9 (3–21)
28-day mortality, *n* (%)	17 (50)
90-day mortality, *n* (%)	21 (62)
1-year mortality, *n* (%)	22 (65)

### ADVOS treatment settings

A total of 102 ADVOS treatments were performed with a median number of 2 (range 1–9) per patient. Median duration of treatment was 17.5 (IQR 11–23) hours per session. Median blood flow rate was 100 ml/min, median concentrate flow was 160 ml/min and median ultrafiltration rate was 100 ml/h. Detailed data regarding blood and concentrate flows, ultrafiltration rate and adjusted dialysate pH are shown in Additional file 1: Table S1.

### Elimination of water-soluble and protein-bound substances

Treatment with ADVOS resulted in a significant decrease in bilirubin levels (− 17.0%; IQR: − 27.8, 0.0), serum creatinine (− 7.1%; IQR: − 26.8, 6.7), BUN (− 17.6%; IQR: − 44.4, 0.0) and ammonia (− 16.4%; IQR: − 36.4, 8.5) levels on a reduction ratio per-session basis (100% × [(pre-treatment value − post-treatment value)/pre-treatment value]). Detailed detoxification data of water-soluble substances and bilirubin in the initial treatment session is presented in Table 2. The reduction ratio elimination rate of bilirubin was concentration dependent as illustrated in Additional file 1: Table S2.

**Table 2:** Elimination of water-soluble substances and bilirubin during the first ADVOS treatment of each patient

	Before ADVOS (mg/dl)	After ADVOS (mg/dl)	Relative elimination for each ADVOS treatment (%)	Rate of treatments showing a reduction of serum levels (%)
Creatinine (mg/dl)	1.65 (1.15, 2.36)	1.13** (0.87, 1.82)	− 24% (− 49%, − 5%)	69
BUN (mg/dl)	28 (17, 44)	16** (11, 23)	− 48% (− 62%, − 17%)	84
Ammonia (µmol/l)	65 (58, 87)	58** (45, 72)	− 19% (− 60%, − 8%)	91
Bilirubin (mg/dl)	4.5 (0.9, 19.1)	4.1** (0.9, 12.6)	− 20% (− 34%, − 4%)	76

Median (IQ25, IQ75). Non-parametric paired Wilcoxon test

**p < 0.01. Median treatment duration 18.5 h (range 8.25, 22.0)

### Acid–base status

**Fig. 2:**
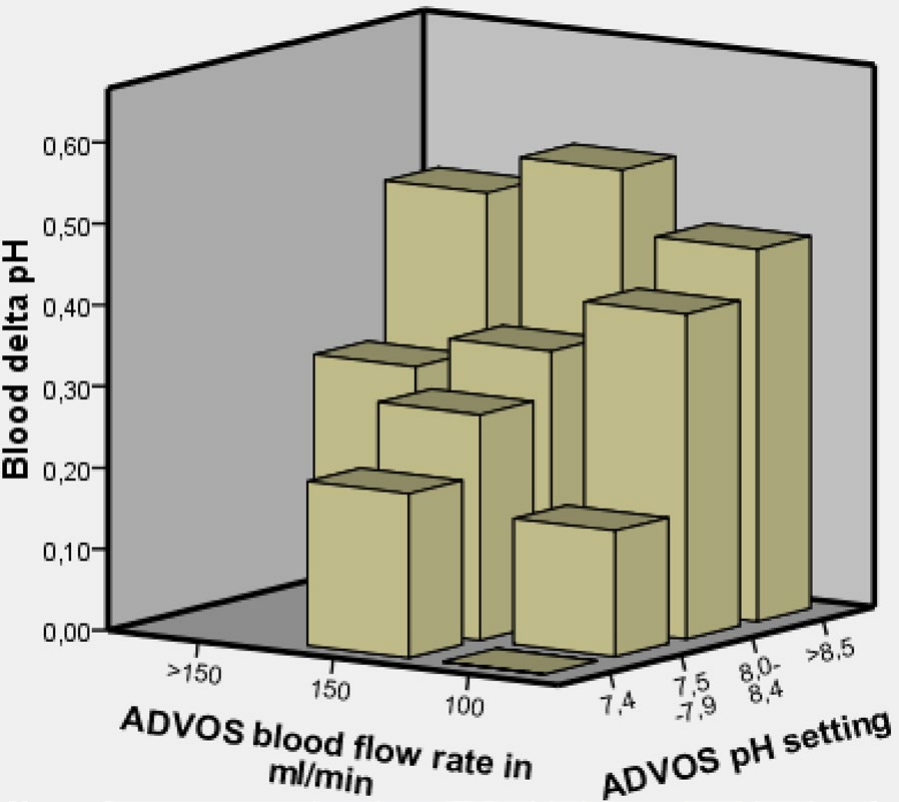
Dialysate blood flow rate, dialysate pH setting of the ADVOS device impact the pH-difference between inlet and outlet (delta pH)

Overall, a significant improvement for blood pH, HCO_3_^−^ and PaCO_2_, could be achieved during treatment as illustrated in Table 3 and Additional file 1: Table S8. Data obtained from consecutive blood samples in the inlet and outlet of the dialyzers highlighted that pCO_2_ reduction and blood pH increase during ADVOS treatment depended mainly on 2 variables: dialysate acid–base composition (which is adjusted based on the dialysate pH set by the treating physician) and blood flow rate of the device. While blood flow correlated specially with pCO_2_ reduction, dialysate pH setting correlated significantly with blood pH, HCO_3_^−^ and pCO_2_ changes, as demonstrated in Fig. 2, Additional file 1: Figures S1, S2 and Table S6.

**Table 3:** Blood gas parameters prior to and immediately after the first ADVOS treatment

	All (*n* = 34)		ARDS (*n* = 10)		Severe metabolic acidosis (*n* = 11)	
	Before	After	Before	After	Before	After
Blood gas						
Blood pH	7.29 (7.19, 7.35)	7.40 (7.32, 7.46)**	7.21 (7.11, 7.29)	7.40 (7.33, 7.48)**	7.19 (7.09, 7.19)	7.40 (7.33, 7.45)**
HCO_3_^−^ (mmol/l)	19.0 (17.5, 23.1)	22.8 (20.0, 27.4)**	24.1 (18.6, 26.7)	32.5 (28.1, 39.6)**	15.4 (13.8, 16.8)	20.4 (18.0, 24.3)**
PaCO_2_ (mmHg)	40.6 (32.9, 60.8)	36.8 (31.6, 43.6)**	68.8 (58.4, 73.2)	49.5 (42.3, 56.1)**	37.5 (31.0, 42.9)	36.8 (32.5, 40.6)
PaO_2_ (mmHg)	81.1 (65.9, 97.8)	78.6 (73.4, 93.2)	73.0 (65.0, 83.3)	84.7 (74.8, 96.9)	88.4 (61.5, 108.2)	77.6 (65.1, 84.2)
Base excess (mmol/l)	− 6.7 (− 9.3, − 1.6)	− 1.5 (− 6.6, 3.1)**	− 0.4 (− 7.0, 2.8)	7.8 (1.6, 15.5)**	− 12.4 (− 14.9, − 10.5)	− 4.9 (− 8.5, 0.9)**
Lactate (mmol/l)	2.20 (1.15, 9.40)	2.50 (1.50, 8.15)	1.90 (0.85, 3.45)	1.95 (1.75, 2.55)	9.60 (5.85, 12.60)	7.40 (5.10, 10.15)
SID (mEq/l)	31.4 (27.2, 35.4)	32.8 (27.5, 39.0)	38.7 (31.2, 40.7)	41.5 (33.4, 47.2)	23.2 (21.3, 25.7)	28.2 (24.6, 34.3)**

Apart from the summary of all treatment sessions, Table 3 illustrates the blood gas parameters of two subgroups (i.e., patients with ARDS and patients with severe metabolic acidosis immediately prior to ADVOS. Median (IQ25, IQ75). Non-parametric paired Wilcoxon test. *p < 0.05, **p < 0.01

The effects of ADVOS on the acid–base system are presented more in detail in two subgroups which are illustrated in the following sections: patients with ARDS and patients with severe metabolic acidemia.

#### ARDS

Twenty-six treatments were performed in ten patients requiring invasive mechanical ventilation and suffered from moderate (*n* = 6) or severe (*n* = 4) ARDS, AKI and liver failure. Median pH prior to the first albumin dialysis was 7.21 (7.11–7.29) and median PaCO_2_ was 69 (IQR: 59–74) mmHg. During the first ADVOS treatment, systemic blood pH levels and PaCO_2_ levels improved to 7.40 (IQR: 7.33–7.48) and to 54 mmHg (IQR: 49–62), respectively (median dialysate pH setting of 8.8 (IQR: 8.2–8.9), driving pressure could be reduced significantly) (Table 3 and Additional file 1: Table S3).

#### Severe metabolic acidosis

Eleven patients suffered from severe metabolic acidemia. Nine patients were mechanically ventilated, and all patients required vasopressor therapy. Six patients were on continuous renal replacement and relocated to ADVOS therapy for refractory acidosis and progressive MOF. Mean lactate levels in these patients were 10.4 ± 7.1 mmol/l prior to initiation of ADVOS. Detailed treatment settings are shown in Additional file 1: Table S1.

pH (7.19 vs. 7.40), HCO_3_^−^ (15.4 vs. 20.4 mmol/l) and base excess (− 12.4 vs. − 4.9 mEq/l) improved significantly in this subgroup of patients during the first treatment with ADVOS, while PaCO_2_ remained constant (37.5 mmHg vs. 36.8 mmHg). A significantly increase of the SID was also observed in this group (23.2 vs. 28.3 mEq/l), mainly due to chloride reduction (Table 3 and Additional file 1: Table S3). The median dialysate pH setting employed during these treatments was 8.5 (IQR: 8.4–8.8). The median duration to normalization of pH was 6 (IQR 3–12) hours. All patients in this group received a single treatment except for one that was treated twice (Additional file 1: Table S8).

### Hemodynamic and respiratory implications of ADVOS treatment

In patients with ARDS, improvements in pH and PaCO_2_ during ADVOS treatments allowed a reduction of driving pressure and maximal inspiratory pressure (Additional file 1: Table S3). Improvement of driving pressure was observed in up to 75% of treatment sessions and was related to baseline driving pressure prior to ADVOS treatment (Additional file 1: Table S4). In addition, there was a trend towards reduced tidal volume and minute ventilation following ADVOS despite the reduction of respiratory support.

Hemodynamics improved significantly during treatment with the albumin dialysis device (Additional file 1: Table S3). Norepinephrine (NE) could be significantly reduced (0.444 vs. 0.375 µg/kg/min; *p* < 0.01) and mean arterial blood pressure (MAP) (69 vs. 74 mmHg, *p* > 0.05) improved following ADVOS treatment. NE could be reduced in 73% of all treatments and in all patients requiring doses < 0.100 µg/kg/min. No NE was required in 43% after ADVOS treatments. Detailed information can be shown in Additional file 1: Table S5.

### Safety and outcome

Treatments were well tolerated, electrolyte levels remained within physiological ranges (Additional file 1: Table S3) and non-significant reduction in the median platelet count (74/nl vs. 60/nl) was observed. Safety data are illustrated in Additional file 1: Table S7. Major bleeding complications were observed in 3 patients during ADVOS treatment. In detail, all 3 patients suffered from cirrhosis with ACLF grade 3 (one patient with 6 organ failure, two with 5 organ failures according to CLIF-SOFA score). One patient with variceal hemorrhage as admission diagnosis developed new variceal hemorrhage during ADVOS with heparin anticoagulation and had successful endoscopic band ligation. The two other patients had diffuse hemorrhage that was already present prior to ADVOS treatment. None of these bleeding complications appeared to be related to ADVOS treatment.

Median length of the ICU stay was 9 days (IQR: 3–22) and the 28- and 90-day, ICU and hospital mortality rates were 50%, 62% 53% and 62%, respectively (Table 1).

## Discussion

The present study reports the feasibility and safety of ADVOS to eliminate water-soluble and protein-bound substances and to significantly support the correction of severe acid–base abnormalities in critically ill patients suffering from MOF.

The principle of the ADVOS therapy is based on a recirculating customizable albumin dialysate. The recycling of the dialysate by physicochemical methods has several consequences. First, since no further albumin addition is needed, treatments can be performed up to 24 h with 200 ml of 20% albumin and low blood flow rates (in our study: 100 ml/min, IQR: 100-150 ml/min), which might facilitate a detoxification of all compartments and an adequate ultrafiltration [41–43]. Second, the modification of pH within the ADVOS multi circuit (Fig. 1) contributes to the release of protein-bound toxins from albumin and its corresponding convective filtration in addition to filtration of water-soluble substances. Furthermore, the possibility to set a customized dialysate pH and with it, modify the acid–base composition of the dialysate permits acid–base control depending on the needs of the patient.

We observed an improvement of hemodynamic parameters like vasopressor dosage and degree of respiratory support like driving pressure during ADVOS therapy. Vasopressors were needed in 74% of the patients before ADVOS treatment. We want to emphasize that these findings are associations which still lack proof of causality. Future studies should clarify whether the reduction of norepinephrine during ADVOS (Table 4 and Additional file 1: Table S3) might be the consequence of improvement and normalization of severe acidemia and if elimination of vasodilatory substances might play an additional role as demonstrated in previous studies [44, 45]. Furthermore, ADVOS significantly reduced water-soluble substances like creatinine, BUN and ammonia. The degree of elimination of these substances was concentration dependent. A reduction of creatinine and BUN could be expected as with other dialysis procedures. Future studies should assess the impact of ADVOS on clinical improvement of hepatic encephalopathy, as shown by other albumin dialysis devices such as MARS [46].

**Table 4:** Illustrations of the differences of post-therapy minus pre-therapy parameters using a random effects linear regression model

Parameter	Pre (mean ± SD)	Post (mean ± SD)	Diff (95% CI)	*p*-value
Bilirubin (mg/dl)	9.7 ± 7.7	7.7 ± 6.1	− 1.9 (− 2.7 to − 1.1)	< 0.001
Creatinine (mg/dl)	1.5 ± 1.1	1.2 ± 0.7	− 0.4 (− 0.6 to − 0.1)	0.002
Ammonia (µg/ml)	59.8 ± 21.6	53.6 ± 27.9	− 6.8 (− 13.3 to − 0.3)	0.039
INR	1.71 ± 1.05	1.71 ± 1.06	− 0.003 (− 0.23 to 0.22)	0.977
Lactate (mmol/l)	4.4 ± 5.5	4.5 ± 6.7	0.5 (− 0.8 to 1.7)	0.69
Na (mmol/l)	139 ± 5	138 ± 5	− 1.4 (− 2 to − 0.7)	< 0.001
K (mmol/l)	4.3 ± 0.3	4.3 ± 0.4	0.01 (− 0.08 to 0.12)	0.747
Cl (mmol/l)	107 ± 5	105 ± 5	− 1.8 (− 2.7 to − 0.9)	< 0.001
pH	7.33 ± 0.9	7.40 ± 0.81	− 0.06 (0.03 to 0.08)	< 0.001
HCO_3_^−^ (mmol/l)	23.9 ± 5.3	26.2 ± 6.8	2.1 (1.1 to 3.2)	< 0.001
CO_2_ (mmHg)	47.3 ± 15.3	42.4 ± 11.1	− 4.5 (− 6.5 to − 2.5)	< 0.001
PaO_2_/FiO_2_	215 ± 113	205 ± 112	− 9 (− 36 to 18)	0.495
Driving pressure (mbar)	15 ± 5	14 ± 3	− 1.1 (− 2.3 to 0.1)	0.078
PEEP (mbar)	10 ± 3	10 ± 3	0.1 (− 0.6 to 0.7)	0.873
Tidal volume (ml)	430 ± 171	414 ± 139	− 17 (− 46 to 13)	0.268
Respiratory rate (per min)	25 ± 5	24 ± 5	− 0.5 (− 1.7 to 0.7)	0.424
MAP (mmHg)	74 ± 17	75 ± 16	0.7 (− 2.8 to 4.2)	0.697
Heart rate (per min)	92 ± 22	86 ± 18	− 5.2 (− 8.4 to − 2)	0.002
Norepinephrine (µg/kg/min)	0.49 ± 1.25	0.39 ± 1.26	− 0.1 (− 0.15 to − 0.05)	< 0.001

In patients treated with ADVOS, a significant reduction of serum bilirubin was observed (Tables 2, 4). Bilirubin is not only a marker of liver disease. New onset of jaundice is well known to contribute to new onset of acute kidney injury and increased rates of infection as demonstrated in a prospective clinical study [47]. An increase of bilirubin 5 times above the upper limit of normal was identified as an independent risk factor for the development of cholemic nephropathy in clinical and autopsy studies [48–50]. In this study, 53% of the patients ended with bilirubin levels < 6 mg/dl. The degree of bilirubin detoxification by ADVOS is quite comparable to other available albumin dialysis [12, 13, 24].

Interestingly, due to the acid–base composition change achieved when a high dialysate pH was set in treatment of acidosis, the ADVOS procedure supported normalization of systemic blood pH with low blood flow rates (100–200 ml/min) within 6 h, even in cases that were refractory to conventional renal replacement therapies [51]. Furthermore, a correlation between dialysate pH set and pCO_2_ reduction of up to 40 mmHg (− 23 mmHg, IQR: − 37, − 7) between the inlet and the outlet of the dialyzer could be observed. In blood, CO_2_ is mainly converted to HCO_3_^−^ and H^+^. A high dialysate pH allows a concentration gradient for H^+^ between blood and dialysate. Moreover, the dialysate contains bicarbonate levels around 20–24 mmol/l, which allows a further gradient for HCO_3_^−^. Consequently, a reduction in pCO_2_ through the removal of HCO_3_^−^ and the compensation of acidosis can be achieved with ADVOS. This can further be explained in terms of a quantitative Stewart approach, based on strong ion difference changes. This is only possible due to an increased buffering capacity of the dialysate thanks to albumin, as this can protonate its imidazole side chain [52, 53], allowing an adequate dialysate acid–base composition adjustment at each set dialysate pH. This pCO_2_ reduction together with the regulation of metabolic acid–base balance was responsible for an improvement of acidosis, as already shown in vitro. We observed a significant reduction of driving pressure during ADVOS treatment in patients with ARDS. We want to emphasize once again that these findings are only associations which still lack proof of causality. Future studies should assess the clinical impact of this alteration in a larger cohort of patients and aim at the proof of causality [54].

A lower mortality rate than expected could be observed in our cohort of patients with advanced stages of MOF treated with ADVOS. Recently, mortality rates > 80% were reported in critically ill patients with SOFA-scores ≥ 17 [55]. In contrast, 28- and 90-day mortality rates in our cohort (with a median SOFA score of 17) were 50% and 62%, respectively. However, this difference can be at least in part be explained by the fact that not all patients in our cohort suffered from sepsis. Moreover, treatment with ADVOS was safe. The number of adverse events were comparable to other studies assessing conventional renal replacement systems in critically ill patients as illustrated in Additional file 1: Table S7 [33, 56].

There are several limitations of our study. First, this is a single-center study with a rather small number of patients. However, it is the first study investigating the ADVOS device in patients with severe MOF. Second, we report data of a non-homogeneous population with AKI and ALF or ACLF with high SOFA score. Therefore, results of this study might not be applicable to other group of patients with single-organ disorders or better prognosis. In this regard, small pilot studies in specific populations that could benefit from this therapy followed by a larger randomized controlled trial are needed for a more detailed assessment of ADVOS.

## Conclusion

Our results demonstrate that the ADVOS device can eliminate water-soluble and protein-bound substances significantly by a new physicochemical method. Furthermore, the device interacts significantly in severe acid–base abnormalities. Future studies should assess its impact in patients with multiple organ failure where a combination of kidney failure and/or liver failure and/or lung failure is present.

## Supplementary information

**Additional file 1: Table S1.** ADVOS treatment parameters. Subgroup analysis. Median (IQR25, IQR75). **Table S2.** Bilirubin elimination in each ADVOS-session depending on bilirubin-levels prior to treatment. Median (IQ25, IQ75). **Table S3.** Ventilation, hemodynamic and electrolytes directly before and after ADVOS treatments. Subgroup analysis: All, ARDS and severe metabolic acidosis. Median (IQ25, IQ75). Non-parametric paired Wilcoxon test. *p < 0.05, **p < 0.01. **Table S4.** Driving pressure variation in each treatment depending on the value before ADVOS treatment among mechanically ventilated patients. Median (IQ25, IQ75). **Table S5.** Norepinephrine (NE) dose variation in each treatment depending on the dose before ADVOS treatment among patients requiring vasopressors. Median (IQ25, IQ75). **Table S6.** Spearman rank correlation of ADVOS blood flow rate and ADVOS pH setting to patients’ delta pH, pCO_2_ and HCO_3_^−^. Delta means the difference of patients’ blood parameters of pH, pCO_2_ and HCO_3_^−^ between the inlet and the outlet of the ADVOS system. r, Spearman correlation coefficient. **Table S7.** Adverse events during ADVOS-treatment. **Table S8.** Blood gas parameters prior to and immediately after each ADVOS treatment. Apart from the summary of all treatment sessions, this table illustrates the blood gas parameters of two subgroups (i.e., patients with ARDS and patients with severe metabolic acidosis immediately prior to ADVOS. **Table S9.** Impact of session duration of ADVOS on several parameters. The median duration of ADVOS treatment (17.5 h) was chosen as cut-off. **Figure S1.** Variation in pCO_2_ and HCO_3_^−^ between the inlet and the outlet of the dialyzer at different dialysate pH settings during ADVOS treatments. Data are stratified according to the dialysate pH setting being employed at the time of blood sampling. **Figure S2.** Variation in blood pH between the inlet and the outlet of the dialyzer at different dialysate pH settings during ADVOS treatments. Data are stratified according to the dialysate pH setting being employed at the time of blood sampling.

## Abbreviations

ACLF: acute-on-chronic liver failure
ADVOS: Advanced Organ Support System
AKI: acute kidney injury
ALF: acute liver failure
ARDS: acute respiratory distress syndrome
AT III: antithrombin III
BUN: blood urea nitrogen
CiCa: calcium citrate
CO_2_: carbon dioxide
ECOS: extracorporeal organ support
HCO_3_^−^: bicarbonate
HLI: hypoxic liver failure
ICU: intensive care unit
ICU-LOS: intensive care unit length of stay
IQR: interquartile range
MARS: molecular adsorbent recirculating system
MOF: multiple organ failure
NA: noradrenalin
Na_2_CO_3_: sodium carbonate
NaHCO_3_: sodium bicarbonate
PaCO_2_: arterial partial pressure of carbon dioxide
pCO_2_: partial pressure of carbon dioxide
P (v–a) CO_2_: venous-to-arterial CO_2_ tension difference
*Q*_B_: blood flow
*Q*_D_: dialysate flow
*Q*_C_: concentrate flow
ROC: receiver operating characteristic
SAPS II: Simplified Acute-Physiology Score II
SOFA: Sepsis-Related Organ Failure Assessment
SPAD: single-pass albumin dialysis
UFH: unfractionated heparin
