# Supplementary material for: Advanced organ support (ADVOS) in the critically ill: first clinical experience in patients with multiple organ failure
Source: Ann Intensive Care. 2020 Jul 16;10:96. doi: 10.1186/s13613-020-00714-3 (PMC7364697; doi:10.1186/s13613-020-00714-3)

table S1. ADVOS treatment parameters during the first ADVOS treatment in each patient. Subgroup analysis. Median (IQR25, IQR75).

|  | **All (n=34)** | **ARDS (n=10)** | **Severe metabolic acidosis (n=11)** |
| --- | --- | --- | --- |
| **Duration (h)** | 18.5  (8.25, 22.0) | 22.5  (16.5, 23.75) | 17.0  (7.5, 20.0) |
| **Blood flow (ml/min)** | 100  (100, 150) | 150  (113, 225) | 100  (100, 160) |
| **Concentrate flow (ml/min)** | 160  (160, 320) | 160  (160, 160) | 320  (160, 320) |
| **Dialysate pH setting** | 8.3  (7.5, 8.7) | 8.8  (8.2, 8.9) | 8.5  (8.4, 8.8) |
| **UF rate (ml/h)** | 0  (0, 168) | 105  (0, 213) | 0  (0, 165) |
| **Dialysate Temperature (°C)** | 28.0  (28.0, 29.5) | 28.5  (28.0, 30.0) | 28.0  (28.0, 28.5) |

table S2. Bilirubin elimination in each ADVOS-session depending on bilirubin-levels prior to treatment. Median (IQ25, IQ75).

| **Bilirubin before ADVOS (mg/dl)** | **Relative elimination for each ADVOS treatment (%)** | **Treatments were level reduction occurred (%)** |
| --- | --- | --- |
| **<6** | 0.0 (-20.0, 28.8) | 38 % |
| **6-12** | -10.8 (-19.7, -4.7) | 86 % |
| **>12** | -23.0 (-30.5, -17.4) | 97 % |

table S3. Ventilation, hemodynamic and electrolytes directly before and after the first ADVOS treatment in each patient. Subgroup analysis: All, respiratory failure and severe metabolic acidosis. Median (IQ25, IQ75). Non-parametric paired Wilcoxon test. *p<0.05, **p<0.01

|  | **All (n=34)** | | **ARDS (n=10)** | | **Severe** **metabolic acidosis (n=11)** | |
| --- | --- | --- | --- | --- | --- | --- |
|  | before | after | before | after | before | after |
| **Ventilation** |  |  |  |  |  |  |
| **FiO_2_ (%)** | 45  (30, 60) | 50  (30, 65) | 70  (60, 85) | 60  (50, 80) | 40  (30, 58) | 60  (45, 85)** |
| **PaO_2_/FiO_2_** | 170  (103, 327) | 155  (114, 256) | 102  (84, 120) | 130  (114, 244) | 136  (105, 244) | 122  (76, 153) |
| **PEEP (mbar)** | 10  (8, 14) | 10  (9, 14) | 14  (9, 15) | 11  (8, 15) | 11  (9, 14) | 14  (10, 15) |
| **Pinsp (mbar)** | 26  (25, 31) | 26  (25, 29)* | 34  (31, 36) | 28  (27, 30)** | 26  (25, 28) | 28  (25, 30) |
| **Driving Pressure (mbar)** | 16  (13, 18) | 15  (14, 17) | 20  (18, 23) | 16  (15, 17)** | 17  (16, 17) | 15  (14, 17) |
| **TV (ml/min/kg)** | 5.8  (4.8, 6.3) | 5.7  (4.5, 6.1) | 5.7  (4.1, 5.9) | 4.8  (4.4, 6.0) | 6.0  (4.9, 6.5) | 5.9  (4.3, 6.1) |
| **Respiratory rate**  **(breath/min)** | 24  (21, 27) | 24  (21, 27) | 27  (24, 29) | 28  (23, 29) | 24  (24, 25) | 24  (22, 26) |
| **Minute Ventilation**  **(l/min)** | 10.5  (8.2, 12.0) | 10.0  (7.7, 12.5) | 10.4  (9.2, 11.1) | 9.0  (7.7, 11.7) | 11.1  (9.6, 12.6) | 10.1  (10.0, 12.6) |
| **Vital signs** |  |  |  |  |  |  |
| **MAP (mmHg)** | 69  (59, 82) | 74  (60, 82) | 67  (63, 72) | 69  (66, 73) | 66  (57, 90) | 68  (60, 83) |
| **Heart Rate (bpm)** | 86  (71, 110) | 93  (71, 99) | 91  (85, 96) | 87  (64, 94) | 84  (77, 131) | 84  (75, 98) |
| **Noradrenalin max. dose (µg/kg/min)** | 0.444  (0.180, 0.800) | 0.375  (0.141, 0.750)** | 0.444  (0.320, 0.629) | 0.170  (0.142, 0.375)** | 0.600  (0.317, 0.836) | 0.480  (0.342, 0.830) |
| **Electrolytes** |  |  |  |  |  |  |
| **Na^+^**  **(mmol/l)** | 141  (136, 143) | 138  (135, 140)** | 142  (139, 143) | 139  (135, 143) | 139  (138, 142) | 138  (135, 140) |
| **Cl^-^**  **(mmol/l)** | 110  (106, 114) | 106  (103, 110)** | 108  (103, 109) | 102  (97, 105)** | 110  (109, 116) | 107  (104, 109)** |
| **K^+^**  **(mmol/l)** | 4.1  (3.9, 4.4) | 4.4  (4.0, 4.6) | 4.5  (4.2, 4.7) | 4.1  (4.0, 4.2) | 4.3  (3.9, 4.8) | 4.5  (4.2, 5.0) |
| **Calcium total (mmol/l)** | 2.12  (1.98, 2.21) | 2.01  (1.94, 2.20)* | 1.98  (1.97, 2.03) | 2.06  (1.98, 2.26) | 2.05  (2.01, 2.15) | 2.04  (1.87, 2.18) |
| **Ca^2+^**  **(mmol/l)** | 1.20  (1.13, 1.25) | 1.18  (1.09, 1.21)** | 1.13  (1.09, 1.23) | 1.14  (1.08, 1.21) | 1.14  (1.05, 1.27) | 1.10  (1.02, 1.21) |
| **Mg^2+^**  **(mmol/l)** | 0.84  (0.73, 0.98) | 0.87  (0.84, 0.92) | 0.81  (0.74, 0.87) | 0.86  (0.85, 0.87) | 0.97  (0.75, 1.00) | 0.90  (0.83, 0.97) |
| **Phosphate**  **(mmol/l)** | 1.11  (0.73, 1.69) | 1.06  (0.76, 1.47) | 1.28  (0.93, 1.91) | 0.79  (0.77, 1.08)** | 1.12  (0.86, 2.30) | 1.38  (0.91, 2.40) |

table S4. Driving pressure variation in each treatment depending on the value before ADVOS treatment among mechanically ventilated patients. Median (IQ25, IQ75).

| **Driving pressure before treatment (mbar)** | **Relative driving pressure variation for each ADVOS treatment (%)** | **Number of treatments** | **Treatments with reduction of driving pressure (%)** |
| --- | --- | --- | --- |
| **<15** | 0.0 (-1.4, 8.33) | 29 | 27.6 % |
| **15-20** | -6.3 (-17.1, 0.0) | 35 | 57.1 % |
| **≥20** | -17.7 (-31.8, -6.8) | 8 | 75.0 % |

table S5. Norepinephrine (NE) dose variation in each treatment depending on the dose before ADVOS treatment among patients requiring vasopressors. Among patients not requiring vasopressors patient before ADVOS treatment, only one needed it after it. Median (IQ25, IQ75).

| **NE dose before treatment (µg/kg/min)** | **Relative NE dose variation for each treatment (%)** | **NE dose reduction during treatment (%)** | **No NE requirement following treatment (%)** |
| --- | --- | --- | --- |
| **≥ 0.001 - 0.100** | -95 (-100, -41) | 100 % | 43 % |
| **≥ 0.100 - 0.500** | -25 (-48, -0.0) | 68 % | 3 % |
| **≥ 0.500** | -20 (-53, 0.0) | 73 % | 6 % |

table S6. Spearman rank correlation of ADVOS blood flow rate and ADVOS pH setting to patients´ delta pH, pCO_2_ and HCO_3_^-^ . Delta means the difference of patients´ blood parameters of pH, pCO_2_ and HCO_3_^-^ between the inlet and the outlet of the ADVOS system. r, Spearman correlation coefficient.

| **Parameter** | **ADVOS blood flow rate (ml/min)** | **p-value** | **ADVOS pH setting** | **p-value** |
| --- | --- | --- | --- | --- |
| **delta pH** | r = 0.421 | <0.001 | r = 0.763 | <0.001 |
| **delta pCO_2_** | r = -0.633 | <0.001 | r = -0.862 | <0.001 |
| **delta HCO_3_^-^** | r = 0.293 | 0.008 | r = 0.915 | <0.001 |

**table S7. Adverse events (AE) in 102 albumin dialysis sessions.**

| **Complication** | **AE present prior to ADVOS** | **AE onset during ADVOS** |
| --- | --- | --- |
| Hemorrhage | 0 | 3 |
| Thrombocytopenia | 68 | 1 |
| Thrombosis | 0 | 0 |
| Hypokalemia | 0 | 0 |
| Hyperkalemia | 7 | 2 |
| Hypophosphatemia | 22 | 0 |
| Hyperphosphatemia | 17 | 0 |
| Hypocalcemia | 43 | 5 |
| Hypercalcemia | 4 | 0 |
| Hypomagnesemia | 9 | 4 |
| Hypermagnesemia | 7 | 0 |
| Cardiac rhythm disorders | 5 | 0 |
| Seizures | 0 | 0 |
| Air embolism | 0 | 0 |
| Catheter insertion complication | 0 | 0 |

Table S8. Blood gas parameters prior to and immediately after each ADVOS treatment. Apart from the summary of all treatment sessions, this table illustrates the blood gas parameters of two subgroups (i.e. patients with ARDS and patients with metabolic acidosis immediately prior to ADVOS. Median (IQ25, IQ75). Non-parametric paired Wilcoxon test. *p<0.05, **p<0.01

|  | **All (n=102)** | | **ARDS (n=26)** | | **Severe metabolic acidosis (n=12)** | |
| --- | --- | --- | --- | --- | --- | --- |
|  | before | after | before | after | before | after |
| **Blood gas** |  |  |  |  |  |  |
| **Blood pH** | 7.36  (7.28, 7.41) | 7.41  (7.36, 7.46)** | 7.30  (7.22, 7.34) | 7.40  (7.33, 7.48) | 7.19  (7.10, 7.19) | 7.40**  (7.35, 7.45) |
| **HCO_3_^-^ (mmol/l)** | 24.4  (18.9, 27.1) | 25.4  (21.6, 29.5) | 28.8  (25.3, 32.2) | 32.2  (28.5, 39.6)** | 14.9  (13.7, 16.5) | 20.9**  (18.2, 24.1) |
| **PaCO_2_ (mmHg)** | 43.6  (35.1, 57.9) | 38.9 **  (32.6, 49.1) | 63.9  (58.6, 74.3) | 54.3  (48.8, 61.5) | 37.0  (31.1, 42.4) | 36.9  (32.5, 39.7) |
| **PaO_2_ (mmHg)** | 78.6  (70.9, 92.0) | 78.9  (71.3, 89.4) | 72.9  (65.4, 83.3) | 77.0  (72.2, 92.4) | 89.6  (61.6, 102.8) | 78.4  (68.2, 83.4) |
| **Base Excess (mmol/l)** | 0.2  (-6.8, 3.4) | 1.4 **  (-3.7, 6.1) | 3.5  (0.8, 8,8) | 9.1  (4.7, 17.3)** | -13.1  (-15.0, -11.1) | -3.4**  (-8.0, 0.8) |
| **Lactate mmol/l)** | 2.15  (1.23, 5.38) | 1.90  (1.33, 5.15) | 1.50  (0.75, 2.60) | 1.65  (0.95, 2.98) | 10.40  (6.8, 12.3) | 6.95  (4.18, 9.28) |
| **SID (mEq/l)** | 35.3  (30.2, 39.8) | 35.1  (29.9, 40.0) | 40.5  (38.6, 43.7) | 42.5  (40.1, 49.0) | 23.0  (21.4, 25.1) | 28.8**  (24.7, 33.8) |

**Table S9**. Impact of session duration of ADVOS on several parameters. The median duration of ADVOS treatment (17.5 hours) was chosen as cut-off.

| Parameter | ADVOS > 17.5 hours | ADVOS ≤ 17.5 hours | p-value |
| --- | --- | --- | --- |
| Delta bilirubin (mg/dl) | -0.07 ± 0.36 | -0.10 ± 0.42 | 0.899 |
| Delta creatinine (mg/dl) | -0.44 ± 0.88 | -0.13 ± 0.42 | 0.053 |
| Delta NH3 (µmol/l) | -11.8 ± 25.5 | -5.3 ± 24.6 | 0.121 |
| Delta INR | -0.0 ± 0.7 | 0.0 ± 0.37 | 0.202 |
| Delta lactate (mmol/l) | -0.16 ± 3.72 | 0.39 ± 3.16 | 0.949 |
| Delta sodium (mmol/l) | -1.4 ± 3.2 | -1.3 ± 2.4 | 0.900 |
| Delta chloride (mmol/l) | -3.0 ± 5.2 | -0.6 ± 4.0 | 0.028 |
| Delta potassium (mmol/l) | 0.1 ± 0.6 | -0.0 ± 0.5 | 0.498 |
| Delta heart rate (per min) | -7.2 ± 14.9 | -3.3 ± 18.3 | 0.187 |
| Delta MAP (mmHg) | 1.8 ± 14.0 | -0.4 ± 21.5 | 0.668 |
| Delta norepinephrine (µg/kg/min) | -0.17 ± 0.31 | -0.03 ± 0.21 | 0.012 |
| Delta PEEP (mbar) | 0.1 ± 2.7 | -0.2 ± 1.3 | 0.800 |
| Delta respiratory rate (per min) | -1.8 ± 4.2 | 0.5 ± 2.9 | 0.011 |
| Delta driving pressure (mbar) | -1.6 ± 4.0 | -0.3 ± 2.2 | 0.162 |
| Delta tidal volume (ml) | -21 ± 112 | -7 ± 66 | 0.630 |
| Delta P/F ratio | 6.4 ± 77.6 | -31 ± 170 | 0.745 |
| Delta pH | 0.07 ± 0.13 | 0.05 ± 0.09 | 0.524 |
| Delta CO_2_ (mmHg) | -3.6 ± 11.7 | - 5.4 ± 8.0 | 0.232 |
| Delta HCO_3_^-^ (mmol/l) | 4.0 ± 7.2 | 1.4 ± 6.4 | 0.049 |

**figure S1.** Variation in pCO2 and HCO3- between the inlet and the outlet of the dialyzer at different dialysate pH settings during ADVOS treatments. Data are stratified according to the ADVOS pH setting being employed at the time of blood sampling.


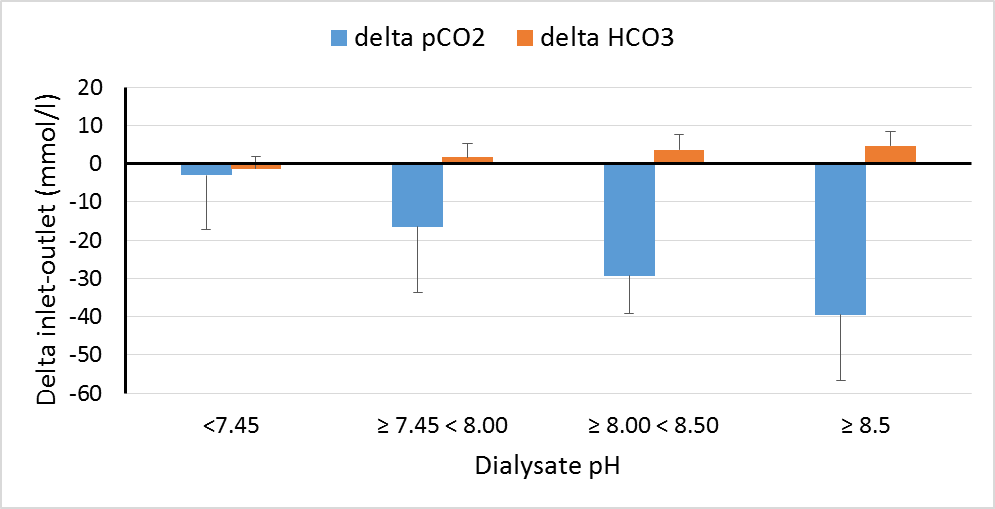


**figure S2.** Variation in blood pH between the inlet and the outlet of the dialyzer at different dialysate pH settings during ADVOS treatments. Data are stratified according to the ADVOS pH setting being employed at the time of blood sampling.


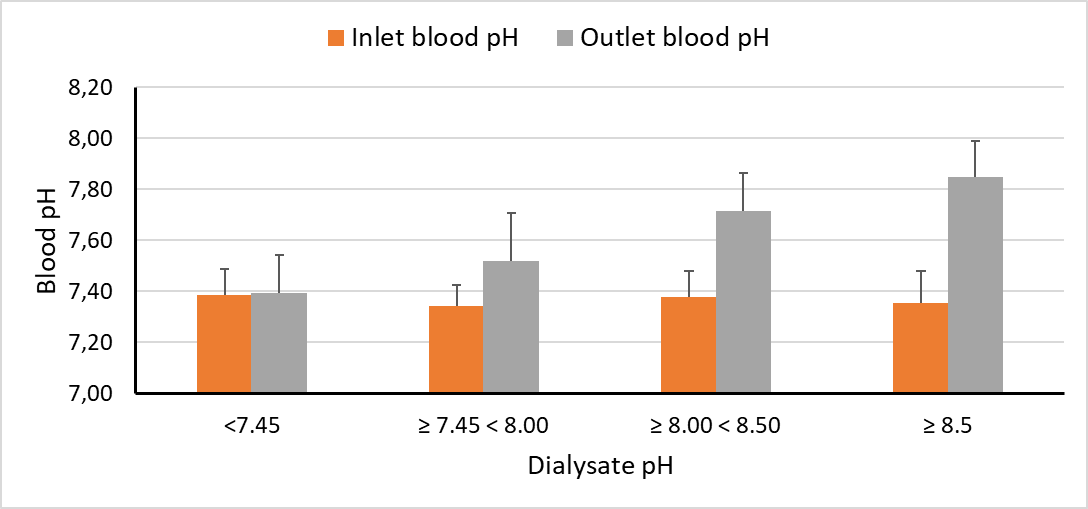

Supplement: Supplementary file 1 — Additional file 1: Table S1. ADVOS treatment parameters. Subgroup analysis. Median (IQR25, IQR75). Table S2. Bilirubin elimination in each ADVOS-session depending on bilirubin-levels prior to treatment. Median (IQ25, IQ75). Table S3. Ventilation, hemodynamic and electrolytes directly before and after ADVOS treatments. Subgroup analysis: All, ARDS and severe metabolic acidosis. Median (IQ25, IQ75). Non-parametric paired Wilcoxon test. *p < 0.05, **p < 0.01. Table S4. Driving pressure variation in each treatment depending on the value before ADVOS treatment among mechanically ventilated patients. Median (IQ25, IQ75). Table S5. Norepinephrine (NE) dose variation in each treatment depending on the dose before ADVOS treatment among patients requiring vasopressors. Median (IQ25, IQ75). Table S6. Spearman rank correlation of ADVOS blood flow rate and ADVOS pH setting to patients’ delta pH, pCO2 and HCO3−. Delta means the difference of patients’ blood parameters of pH, pCO2 and HCO3− between the inlet and the outlet of the ADVOS system. r, Spearman correlation coefficient. Table S7. Adverse events during ADVOS-treatment. Table S8. Blood gas parameters prior to and immediately after each ADVOS treatment. Apart from the summary of all treatment sessions, this table illustrates the blood gas parameters of two subgroups (i.e., patients with ARDS and patients with severe metabolic acidosis immediately prior to ADVOS. Table S9. Impact of session duration of ADVOS on several parameters. The median duration of ADVOS treatment (17.5 h) was chosen as cut-off. Figure S1. Variation in pCO2 and HCO3− between the inlet and the outlet of the dialyzer at different dialysate pH settings during ADVOS treatments. Data are stratified according to the dialysate pH setting being employed at the time of blood sampling. Figure S2. Variation in blood pH between the inlet and the outlet of the dialyzer at different dialysate pH settings during ADVOS treatments. Data are stratified according to the d [file 13613_2020_714_MOESM1_ESM.docx]
